# Dermoscopy of pyogenic granuloma

**DOI:** 10.11604/pamj.2017.27.110.12278

**Published:** 2017-06-13

**Authors:** Amina Kissou, Badr Eddine Hassam

**Affiliations:** 1 Department of Dermatology, Ibn Sina University Hospital, Rabat, Morocco

**Keywords:** Dermoscopy, granuloma, pyogenic

## Image in medicine

A patient aged 58 years, presented with 2 months history of an erythematous, painful and bleeding nodule, after labial herpes (A). The dermoscopic examination confirms the diagnosis of a pyogenic granuloma (B). The dermoscopic criteria are: the reddish homogeneous area (green line) whose color varied from completely red to red with whitish zones which is corresponded to proliferating vessels. A white collarette (yellow line) corresponds to the hyperplastic adnexal epithelium that embraces the lesion at the periphery. The white lines (blue line) like a double rail which is corresponded to a fibrous septa that criss-cross the lobules. And finally ulceration or hemorrhagic crusts (black line) which are frequently observed in pyogenic granuloma.

**Figure 1:**
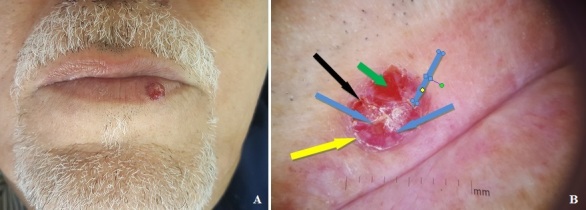
Erythematous bleeding nodule of the lower lip

